# Effectiveness of Platelet-Rich Plasma and Bone Marrow Aspirate Concentrate as Treatments for Chronic Hindlimb Proximal Suspensory Desmopathy

**DOI:** 10.3389/fvets.2021.678453

**Published:** 2021-06-18

**Authors:** Grigorios Maleas, Mahmoud Mageed

**Affiliations:** Tierklinik in Lüsche GmbH, Bakum, Germany

**Keywords:** orthobiologics, horse, platelet-rich plasma, suspensory ligament, bone marrow aspirate, healing

## Abstract

This retrospective study aimed to evaluate the clinical effect of bone marrow aspirate concentrate (BMAC) and leukocyte rich PRP (LR-PRP) compared to horses undergoing controlled exercise alone in horses with >3 months proximal suspensory desmopathy in hindlimbs (HPSD). Nighty-three horses were divided into three groups according to the treatment: a control (*n* = 22), LR-PRP (*n* = 46), and BMAC (*n* = 25) group. Lameness and ultrasound scores were recorded before treatment (T0) and at 6 months (T1) post-treatment. Records horses considered sound at evaluation and level of performance were additionally registered at 12 months (T2) and 18 months (T3) after treatment. The BMAC cytology profiles from 22 horses were also analysed and compared to clinical outcomes. The results at T1 showed that 9% (2/22) of the horses in the control group were sound compared to 59% (25/46) and 84% (21/25) in the LR-PRP and BMAC groups, respectively. Additionally, ultrasound scores at T1 in the BMAC and LR-PRP groups were improved in comparison with the control group (*p* = 0.02). At T2, 68% of the horses in the BMAC group and 39% of the horses in the LR-PRP group had returned to the previous performance level. At T3, a significantly higher percentage of horses in the LR-PRP (43%) and BMAC (72%) group were sound when compared to the control (4.6%) group (*p* = 0.02). Similarly, at T3, significantly more horses of the BMAC (16/25) and of the LR-PRP (15/46) group had returned to the previous or a higher performance level compared to the control (1/22) group (*p* = 0.01). No correlation was found between long-term clinical outcome and cytology profiles in the BMAC group. In conclusion, long-term outcomes of treatment with LR-PRP or BMAC are significantly better than conventional treatment of the hindlimb chronic PSD in horses. Additionally, BMAC yielded better lameness scores than LR-PRP at short- and long-term follow-up.

## Introduction

Desmopathy of the hindlimb proximal suspensory ligament (HPSD) is a common cause of lameness in horses (1, 2). Non-surgical conventional treatments of HPSD include local infiltration with corticosteroids and/or hyaluronan, extracorporeal shock wave therapy (ESWT), and systemic application of NSAIDs in combination with corrective shoeing (1, 3, 4). However, each of these have shown not satisfying long-term results in chronic cases (>3 months) (1–6).

Orthobiologics such as platelet-rich plasma (PRP) and regenerative cells have gained continuous attention due to their potential to stimulate repair mechanisms and reduce the risk of re-injury in tendons and ligaments (7–11). It has been shown that many PRP products contain supraphysiological concentration of growth factors, which seem to have the potential to support ligament regeneration in chronic HPSD based on angiogenesis, cell proliferation and stimulation of extracellular matrix components (7, 8, 12, 13). The use of stem and regenerative cells produced in commercial laboratories have yielded good results in HPSD (14, 15). Nonetheless, there is a lack of research regarding clinical outcomes after regenerative treatment in chronic HPSD cases. Limitations of using cultured stem cell therapies are the high cost and the prolonged preparation methods required. Furthermore, legislation was passed in the European Union restricting the use of stem cells in veterinary medicine (16). Consequently, there is an increasing need for a cost-effective and single-step cell-based treatment.

In comparison with bone marrow aspirate (BMA), BMA concentrate (BMAC) consists of higher numbers of progenitor and nucleated cells, growth factors, cytokines, and platelets (17). Treatment with BMAC has been shown to yield a significantly lower rate of re-injury in human tendinopathies compared with conventional treatments (18). In horses, the anabolic effects of acellular BMA applied to suspensory ligament fibroblasts *ex vivo* have been reported to be higher than those of PRP treatment (19). Additionally, a case series which investigated the use of autologous bone marrow for treating desmopathy of proximal suspensory ligament (PSL) in fore- and hindlimbs reported promising results (*n* = 89/100 were sound) (20). However, to our knowledge, no previous equine studies have investigated the efficiency of BMAC in chronic HPSD. Therefore, the primary objective of the current study was to evaluate the short and long term clinical and ultrasonographic outcomes of ultrasound guided intralesional PRP and BMAC as treatments for chronic HPSD. The secondary objective was to analyse the cytology profile of BMAC and its correlation with the clinical outcome. We hypothesised that BMAC would show better short- and long-term outcomes than PRP, and that both BMAC and PRP would show better results than the control group.

## Materials and Methods

### Patient Recruitment

This present research takes the form of a retrospective longitudinal study design. The medical records of all horses presented at two equine clinics (Tierklinik in Lüsche GmbH, Germany and Heilan Equestrian Club in Jiangsu, China) between 2016 and 2020 were reviewed. The included horses met the following criteria: (1) non-racing sports horses with poor performance due to unilateral, >3 months hind limb lameness localised in the area of PSL [negative low plantar anaesthesia, substantial reduction (>80% determined subjectively) or alleviation of the lameness or poor performance within 10 min after local infiltration of the PSL (2 mL, mepivacaine) (4)]; (2) ultrasonographic abnormalities of the PSL in comparison with the contralateral limb (enlargement, multiple or diffuse reduction in echogenicity, and loss of definition of the dorsal borders) (1, 4); (3) normal radiographic appearance of the proximal third metatarsal bone and the tarsometatarsal and distal intertarsal joints, and negative intra-articular anaesthesia of tarsometatarsal joint (3 mL, mepivacaine, 10 min); (4) no previous treatments before presentation except controlled exercise; (5) clinical and ultrasonographic follow-up after rehabilitation; and (6) records of their performance and orthopaedic clinical evaluation at 12 and 18 months post-treatment. The horses were divided into groups retrospectively based on treatment type: a control, a PRP, and a BMAC group. The choice of the orthobiological treatment was dependent upon clinician's preference. The control group was comprised of horses undergoing controlled exercise alone without intralesional treatments due to owner preference.

Horses were excluded if (1) an additional treatment was applied; (2) a different rehabilitation plan was implemented than that recommended; (3) they withdrew from the sport for a reason unrelated to hindlimb PSL.

### Initial Clinical and Ultrasound Evaluation

**Figure 1:**
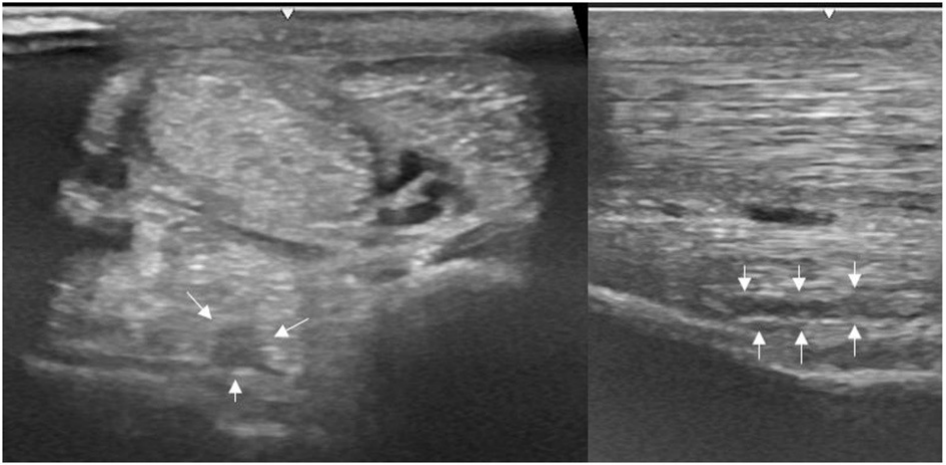
A transverse image of a proximal suspensory ligament grade 1 lesion about 4 cm distal to the head of the MTII. The lesion appears as a focal, well-defined, hypoechogenic area (white arrows) in the ligament occupying <25% of the cross-sectional areal. Longitudinally the lesion is well-demarcated (white arrows), it begins from the insertion and extends distally. In transverse image medial is to the left; in longitudinal image proximal is to the left.

**Figure 2:**
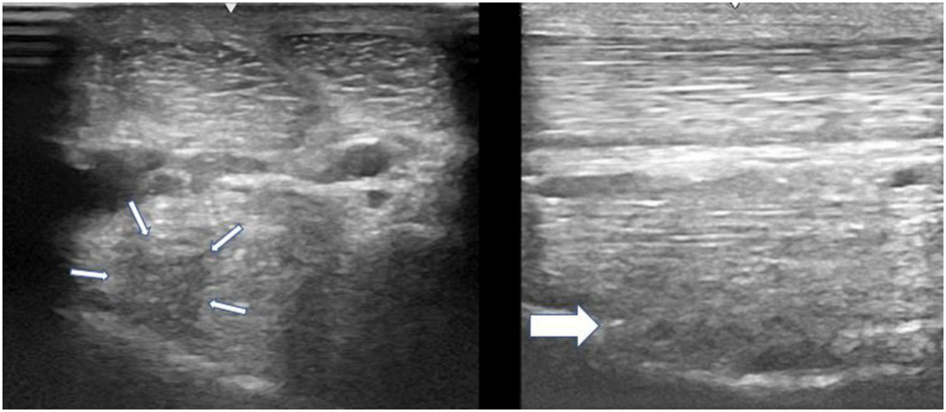
A transverse image of a proximal suspensory ligament grade 2 lesion (small white arrows) about 3 cm distal to the head of the MTII. The character of the lesion is similar to the Figure 1, but with larger cross-sectional area. In the longitudinal image there is a disruption of the fibre pattern (big white arrow) in the dorsal aspect of the ligament. In transverse images medial is to the left; in longitudinal images proximal is to the left.

**Figure 3:**
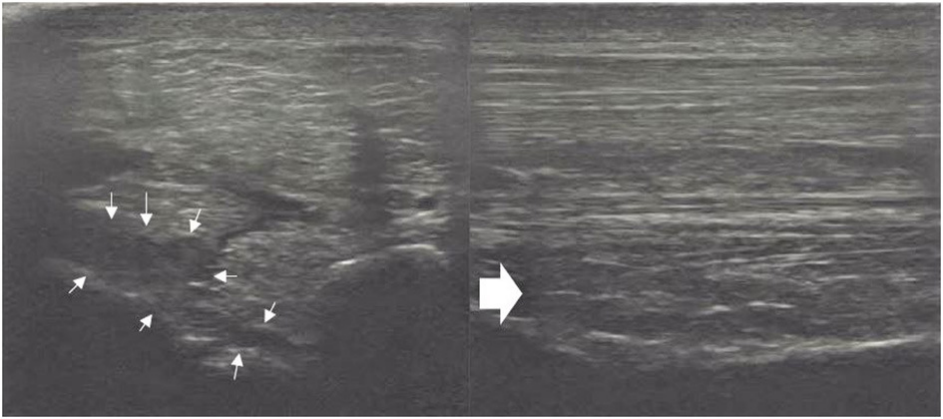
A transverse image of a proximal suspensory ligament grade 3 lesion about 3 cm distal to the head of the MTII and one longitudinal image of proximal metatarsal area. The hypoechogenic lesion extends transversely in the entire dorsal area of the ligament (small white arrows). Longitudinally, more than 50% of the fibre pattern is lost and the dorsal margin of the ligament has poor demarcation. In transverse images medial is to the left; in longitudinal image proximal is to the left.

**Figure 4:**
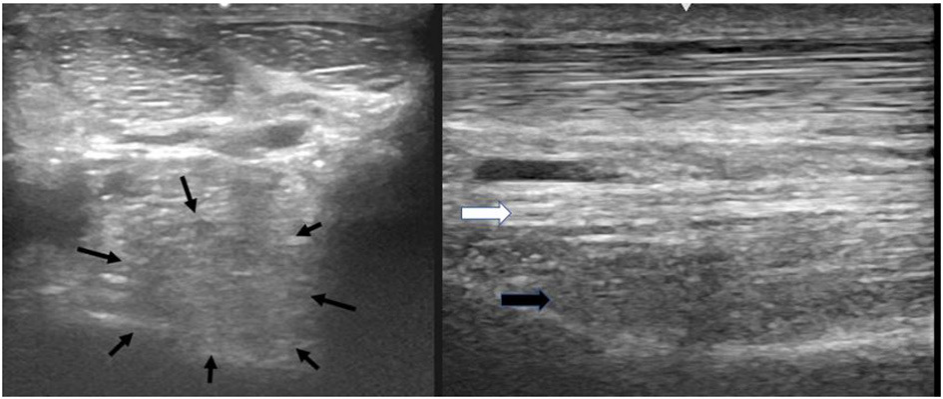
A transverse image of the proximal suspensory ligament about 4 cm distal to the head of MTII with a grade 4 lesion. The majority of the ligament's cross-sectional area (more than 75%) appears diffusely hypoechogenic. In the longitudinal image only a small plantar area of the ligament maintains normal fibre pattern. The dorsal margins of the ligament cannot be distinguished. In transverse images medial is to the left; in longitudinal image proximal is to the left.

A thorough clinical assessment was carried out at presentation day by the first author using a standardised protocol: A detailed anamnesis was initially obtained. Simultaneously, the hooves, limbs, back and neck were evaluated by a short observation and palpation. After that, the horse was walked and trotted in-hand on the straight line and at both reins on circles, on the concrete and soft surface. Additionally, the horse was lunged to assess the trot, the canter, and the transitions from canter to trot. Subsequently, distal and proximal flexion tests were performed. In all cases of hindlimb lameness, the Churchill hock test was also conducted. After the clinical evaluation diagnostic analgesic techniques were performed starting with four-low-point and continuing proximally. After a negative low-four-point the tarsometatarsal joint was blocked first. If the intraarticular diagnostic analgesia was only partially positive (<50% subjectively evaluated), the PSL infiltration was performed the next day. A ridden examination either with the owner or with an experienced rider from the clinic staff was performed if deemed clinically relevant. Lameness was assessed according to the AAEP grading scale (21). The ultrasound evaluation was performed with a linear 8–10 MHz probe, 1–3 days after diagnostic analgesia. The PSL was examined using the standard plantar medial approach for transverse, longitudinal in weightbearing, and non-weightbearing scans (22). Transverse images were captured in standardised locations (about 3, 4, 5, and 6 cm distal to the head of the MTII) in weight- and non-weightbearing scans. The capture of the longitudinal images was performed on a weightbearing limb and by tilting the probe laterally and medially. All ultrasonographic images were graded retrospectively and blindly by the second author. The ultrasonographic grade of the PSL was assigned at the location of maximal severity in weightbearing appearance according to previous literature (9, 14) as follows: 0 = no detectable lesion, 1 = lesion/fibre pattern disruption 5–25% of the total cross-sectional area of the ligament at that location (Figure 1) (lesion in cross-sectional area = LCSA); 2 = 26–50% LCSA (Figure 2); 3 = 51–75% LCSA (Figure 3); 4 = 76–99% LCSA (Figure 4).

### Preparation of PRP

PRP was prepared in a fully enclosed gravitational system in accordance with the manufacturer's instructions (E-PET, Pall Inc., Port Washington, NY, USA). Briefly, after obtaining 55 ml blood from the jugular vein after aseptic preparation and mixing it with 5 mL anticoagulant, 6–8 mL of leukocytes rich (LR) PRP was obtained in ~15 min through gravity flow filtration. This method can concentrate 3.8–4 × platelets and 1.8 × leukocytes above the baseline level in blood (23).

### Preparation of BMAC

All procedures were performed by the first author as previously described (24). Briefly, after sedation, the sternum was clipped 5–10 cm caudal to the olecranon and aseptically prepared. After local anaesthesia of the puncture side, an 11-gauge fenestrated bone marrow biopsy needle (Stryker Spine Inc., Allendale, NJ, USA) and 10-mL syringes were rinsed thoroughly with heparin and then filled with 1,000 USP units of heparin/1 mL BMA. During aspiration, the needle was rotated 90° every 4–5 mL. Following extraction of 30–40 mL of aspirate, the latter was transferred into sterile 10-mL tubes through a 200-μm filter (Sangofix, B.Braun GmbH, Hessen, Germany) to remove particulate matter. The filtrated BMA was centrifugated (1,000 g for 10 min), and the buffy coat with the last 1/4 of the supernatant were carefully extracted with a long spinal 18G needle in a separate tube under sterile conditions and the rest was discharged. This method was adapted and modified from a human BMAC preparation protocol (25). All steps of preparation in human protocol were similar to the protocol of this study, except of using 100 IU heparin/1 ml BMA and the centrifugation's speed was 1,300 g for 10 min. However, 100 IU heparin/1 ml equine BMA caused severe coagulation in the first attempts to prepare equine BMAC. Hence, the filtration was impossible. This limitation could be substantially diminished by using 1,000 IU heparin/1 ml BMA. Additionally, a small pilot study with five horses in the authors' clinic showed that more total nuclear cells can be obtained by a speed of 1,000 g in comparison with 1,300 g. The final product yielded ~4–6 mL of BMAC. For cytology analysis, 1-mL samples of both BMA and BMAC were taken from 22 horses and processed in the clinic's laboratory. The analysis included measurements of white blood cells (WBC), monocyte, platelet, RBC counts, and PCV.

### Treatment, Rehabilitation, Evaluation, and Follow-Up

Prior the preparation of the orthobiological treatment, the tibial nerve was initially anaesthetised with 20 ml of mepivacaine to reduce the discomfort of the patient. During the preparation of BMAC and LR-PRP, the injection site was aseptically prepared. The limb was elevated, and the ultrasound probe was placed transversely. The author used 21 G needles and luer-lock syringes. The needle was inserted lateral to the abaxial margin of the SDF tendon and axial to MTIV. Between 2 and 4 mL were administered under ultrasound guidance as an intralesional injection. In case of extended lesions distally, the probe was repositioned accordingly and the intralesional injections were repeated. Attempt was always made to inject any possible hypoechogenic lesion. After the injections, a bandage was applied, and a 2-day box confinement was instructed. Thereafter, the horses started with walking in-hand for 6 weeks and ridden at a walk for the following 6 weeks at least 45 min twice daily. No turnout in the paddock was allowed. After this period, the referring vet performed a short clinical and ultrasound examination. The ultrasound images were sent to the first author for assessment and the case was discussed together with referring vet. If the horse was then sound and the ultrasound score was reduced at least by one point, a 10-week, gradually increased exercise programme was prescribed, beginning with 2 min of trotting in a straight line at the end of the riding walk. In case of detection of concurrent sacroiliac and/or thoracolumbar back pathology in the initial examination either through physical examination or through diagnostic local analgesia, additional treatments were applied during this period (local corticosteroids injections by the referring vet and/or physical therapies, e.g., chiropractic and dynamic mobilisation). However, if the lameness persisted, the riding walk was extended for another 6–12 weeks and then re-evaluated. If the horse was sound at the end of the rehabilitation period, gradual return to its previous level of activity was permitted. The duration of this period was depended upon the discipline, the previous level of performance and fitness of each horse individually. On average, jumping horses needed about 4–6 weeks and event horses ~8 weeks to reach their previous fitness level. On the contrary, dressage horses were back to normal training 2–4 weeks after the end of the rehabilitation. In case of persistent lameness at the end of the rehabilitation period, the therapy was identified as being a failure and the respective horses were not included in the follow-up anymore. All horses, independent of treatment, underwent foot dorsoplantar (long toe, narrow heels, overgrown heels, and difference in hoof angles) and mediolateral (unilateral overgrown/concave hoof wall) imbalance corrections and wide egg-bar shoe application at the start of rehabilitation. Orthopaedic shoes (egg-bar or straight-bar shoes, wide-toe and narrow-heeled shoes, hind rocking shoes) were also fitted after the return to normal training depending on the pastern and hoof conformation. The control group horses were additionally administered phenylbutazone orally for 10 days. Re-examination was carried out 6 months (T1) post-treatment by the first author and the findings of the lameness exam (AAEP scale) and ultrasound (grading as at T0) score were recorded. A telephone questionnaire was conducted with the referring veterinarians at 12- and 18-months post-treatment to assess the long-term outcome in terms of percentage of horses considered sound at evaluation and performance level.

### Data Analysis

Statistical analyses were performed using commercial software (SPSS 25.0, IBM Munich Centre GmbH, Munich, Germany). The normal distribution was tested using the Sharipo–Wilk Test. The Kruskal–Wallis test was used to compare ultrasound and lameness scores among the groups at presentation (T0), the Wilcoxon single-rank test was applied to compare ultrasound and lameness scores within groups at T0 and T1, and the Mann–Whitney *U*-test was used to compare the ultrasound and lameness scores at T1. Additionally, the Mann–Whitney *U*-test was used to compare the cytology profile of BMAC and the clinical outcome and level of performance at T3. The Chi-squared test was used to compare the significance of percentage of horses considered sound at evaluation and level of performance proportions among the control, LR-PRP and BMAC groups at T2 and T3. The significance level was set at *p* ≤ 0.05.

## Results

Initially, 101 horses met the inclusion criteria, but eight animals were excluded due to additional therapies and failure to follow them up. Table 1 summarises the signalment of the remaining 93 subjects (69 Warmbloods, 3 Thoroughbreds, 4 German riding ponies, and 17 Andalusians horses). There were no significant differences between the groups in terms of age, but the horses in the control group tended to be younger. The baseline lameness and ultrasound scores also did not have any significant difference among the groups. In 43 cases, the main complaint was poor performance, and the riders presented the horses to the clinic because of suspecting back pain. Ridden examination was performed in 61 cases. Dressage horses predominated the study population (*n* = 65). The PSL of the left hindlimb was more commonly affected in the control (*n* = 14) and BMAC (*n* =14) groups, while the right hindlimb was more commonly affected in the LR-PRP group (*n* = 25). No severe side effects or complications were observed to be associated with either the LR-PRP or the BMAC treatments. A temporary, mild swelling at the proximal third of metatarsus at the injection side was observed after the orthobiological treatment (LR-PRP or BMAC) in 52/71 horses (73.2%), which disappeared without any additional treatment within 10 days post-treatment.

**Table 1:** Summary of the horses' signalment and lameness grades at presentation (T0).

		**Control**	**PRP**	**BMAC**
Number of horses		22	46	25
Age median in years (range minimum–maximum)		10 (4–17)	12 (4–21)	11 (6–19)
Gender	Mare	13	12	9
	Gelding	8	27	12
	Stallion	1	7	4
Discipline	Dressage	17	32	16
	Eventing	1	7	6
	Jumping	4	7	3
Breed	Warmbloods	18	34	17
	Thoroughbreds	0	1	2
	German riding ponies	0	3	1
	Andalusians	4	8	5
Lameness T0 median (minimum–maximum)		2 (1–3)	2 (1–3)	2 (1–3)
Lameness T1 median (minimum–maximum)		2 (0–3)	0 (0–3)	0 (0–2)
percentage of horses considered sound at T1 (%)		2 (9)	25 (59)^*^	21 (84)^*^
percentage of horses considered sound at T2 (%)		2 (9)	23 (50)^*^	18 (72)^*^
percentage of horses considered sound at T3 (%)		1 (4.6)	20 (43)^*^	18 (72)^*^
Same or higher level of performance (%) at T2		1 (4.6)	18 (39)^*^	17 (68)^*^
Same or higher level of performance (%) at T3		1 (4.6)	15 (33)^*^	16 (64)^*^

*At six (T1), 12 (T2), and 18 (T3) months post-treatment percentage of horses considered sound at evaluation was documented. Additionally, at T2 and T3 the proportion of the horses of the same or higher level as before the injury was recorded*.

* *p > 0.05*.

**Figure 5:**
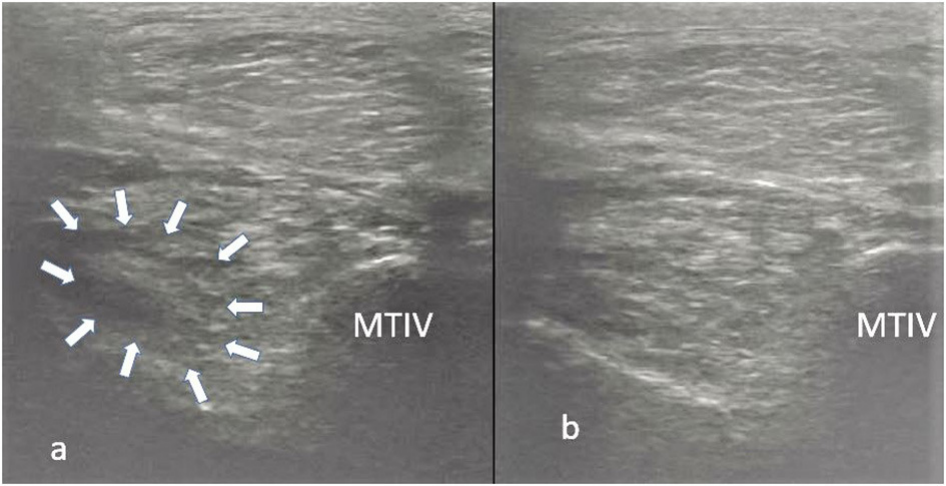
Transverse images of a proximal suspensory ligament about 4 cm distal to the head of the MTII. In panel **(a)** is the lesion (white arrows) before the treatment and in panel **(b)** 24 weeks after the treatment with LR-PRP. The healing is characterised by reduction of the hypoechogenecity of the lesion. This horse remained sound at the 18-month follow-up.

**Figure 6:**
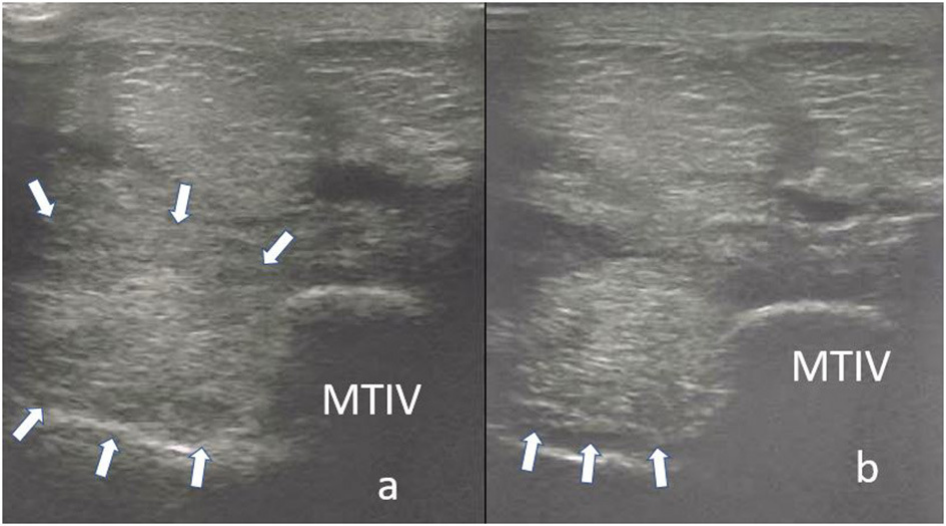
Transverse images of the proximal suspensory ligament about 4 cm distal to the head of the MTII. In panel **(a)**, the ligament appears enlarged with poor demarcation of its dorsal and plantar margins. Additionally, two focal hypoechogenic lesions are present in the dorsal aspect of the ligament. In panel **(b)**, the suspensory ligament 24 weeks after the treatment with BMAC. There is a re-appearance of the dorsal and plantar margins and reduction of the size of the ligament. Moreover, a remnant of the lesions in dorsal aspect is still visible. Nevertheless, the horse was sound 18 months after the treatment.

**Figure 7:**
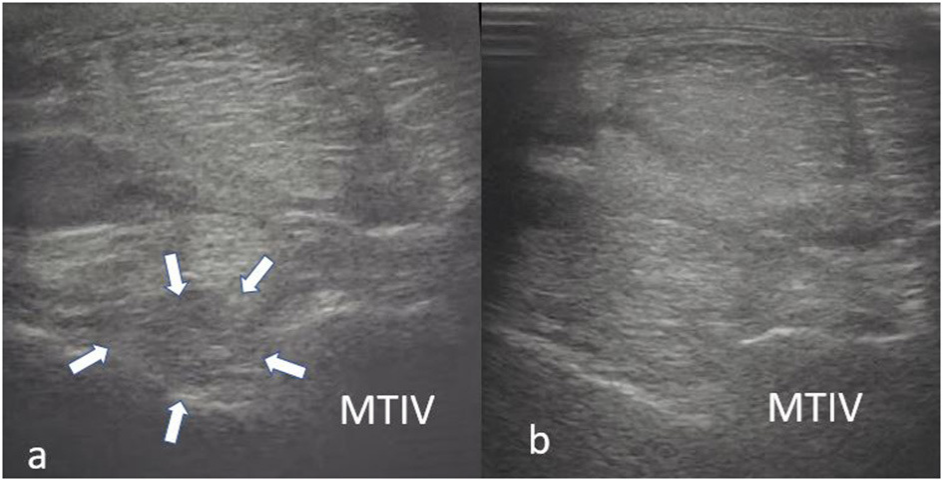
Transverse images of a proximal suspensory ligament about 3 cm distal the head of the MTII. In panel **(a)**, the dorsal, hypoechogenic lesion (white arrows) occupies more than 50% of the cross-sectional area of the ligament. In panel **(b)**, there is an obvious increase in echogenicity of the dorsal aspect of the ligament. This horse was treated with BMAC and was not lame 18 months post-treatment.

**Figure 8:**
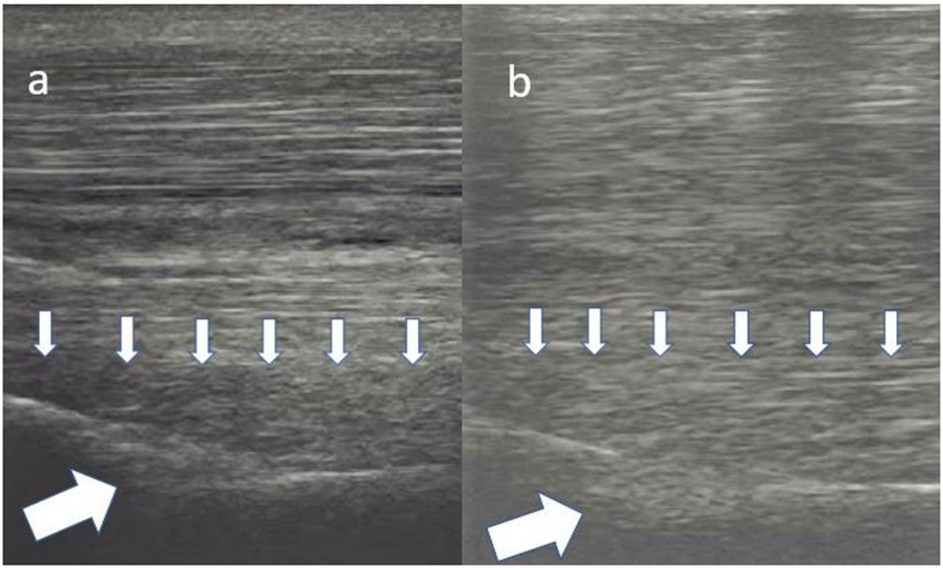
Longitudinal images of a proximal suspensory ligament treated with LR-PRP. The lesion in panel **(a)** is characterised by at least 50% loss of the fibre pattern of the dorsal area of the ligament (small arrows). There is also a hypoechogenic area in the bony contour of the MTII (big white arrow). In panel **(b)**, there is re-appearance of the fibres in the lesion (small arrows). The echogenicity in the plantar contour of MTIII is increased. This horse was sound on evaluation 18 months after the treatment.

No significant difference in either lameness (for all horses median = 2/5 AAEP, range 1/5–3/5 AAEP) or ultrasound score (for all horses median = 2, range 1–4) was found among the three groups at T0 (lameness, *p* = 0.473, ultrasound, *p* = 0.746) (Table 1). At T1, 9% (2/22) of the horses in the control group were sound and the lameness improvement within this group was not significant (*p* = 0.059). In the LR-PRP and BMAC groups 59% (25/46) and 84% (21/25) of the horses were evaluated sound at T1, respectively. The BMAC group showed a significant improvement in lameness score at T1 compared to T0 (*p* = 0.001), with similar results reported in the PRP group (*p* = 0.001). Additionally, both LR-PRP and BMAC groups showed significant lameness improvement compared to the control group at T1 (*p* = 0.007). Furthermore, ultrasound scores were significantly improved at T1 (Table 2) compared with T0 in both the BMAC group (*p* < 0.001) and the LR-PRP group (*p* < 0.001) (Figures 5–8). Additionally, ultrasound scores at T1 in the BMAC and LR-PRP groups were significantly improved in comparison with the control group (*p* = 0.02). At T2, 68% of the horses in the BMAC group and 39% of the horses in the LR-PRP group were observed to have returned to their previous performance level and the difference between these groups was significant (*p* = 0.02). At T3, 43% of the LR-PRP group and 72% of the BMAC group were still sound after treatment, with a significant difference between both groups (*p* = 0.02), whereas this was only the case for one horse in the control group (4.6%). At the same time, 16/25 (64%) in the BMAC and 15/46 (33%) in the LR-PRP group returned to and performed at the same or higher performance level (*p* = 0.01; Table 1). All lameness or recurrent lameness cases in T1, T2, and T3, which were unresponsive to treatment, were due to HPSD. The latter was diagnosed clinically and sonographically either by the referring vet or by the first author. In eight horses, the ultrasound score did not change at T1, in spite of the animals being sound. However, all these 8 horses experienced a recurrence of HPSD within 1 year after treatment.

**Table 2:** Ultrasound scores among the groups at T1.

**Group**	**Lesion 0**	**Lesion 1**	**Lesion 2**	**Lesion 3**	**Lesion 4**
PRP, considered sound/total (%)	4/5 (80)^*^	14/21 (66.6)^*^	2/13 (15.3)^*^	0/7 (0)^*^	0
BMAC, considered sound/total (%)	2/3 (66.6)^*^	15/18 (83.3)^*^	1/3 (33.3)^*^	0/1 (0)^*^	0
Control, considered sound/total (%)	0/0 (0)	1/9 (100)	0/12 (0)	0/1 (0)	0

*Within the parenthesis is the percentage of horses considered sound at the evaluation 18 months after the treatment*.

* *p > 0.05*.

The analysis of cytology profiles of the BMA and BMAC group is presented in Table 3. A significantly higher concentration of WBC, monocytes, and platelets were observed in the BMAC compared to the BMA group (*p* < 0.001). The red blood cell concentration was also significantly reduced (*p* < 0.001). In the BMAC group, there was no significant correlation among cell concentrations and lameness/ultrasound at T1 or return to previous level of performance at T2. Similarly, no significant differences were found at T3 in terms of WBC (*p* = 0.682), monocyte (*p* = 0.892) and platelet (*p* = 0.25) count between horses competing at the same or higher level than pre-injury (*n* = 14) and those which were lame (*n* = 8).

**Table 3:** Cytology profiles of bone marrow aspirate (BMA) and its concentrate (BMAC).

	**BMA**	**BMAC**	**Median relative change**
WBC (×1,000/μL)	23.11 (34.7–15)	86.5 (235.6–54.6)	3.2 (8.9–1.2)^*^
Monocytes (×1,000/μL)	1.95 (0.95–3.9)	7.33 (22.9–3.27)	3.5 (8.9–1.4)^*^
Platelets (×1,000/μL)	54 (570–27)	160 (560–46)	2.4 (7.8–0.7)^*^
PCV (%)	22.7 (78–20)	10.5 (40–2.2)	0.3 (0.9–0.08)^*^
RBC (×1,000/μL)	5.34 (15.7–3.76)	2.78 (8.4–0.4)	0.2 (0.5–0.04)^*^

*All values are presented as median (range maximum–minimum)*,

* *p > 0.05*.

## Discussion

This retrospective controlled study aimed to evaluate the effectiveness of LR-PRP and BMAC as treatments for chronic HPSD compared to controlled exercise alone. To the best of the authors' knowledge, this is the first retrospective evaluation of intralesional BMAC and PRP treatments compared to untreated control in horses with HPSD. The results showed a significant improvement in lameness following treatment with either LR-PRP or BMAC compared to the control group at six- and 12-months post-treatment and are superior to previous studies evaluating controlled exercise alone for chronic HPSD (1, 3). Controlled exercise alone treatment is not one of the authors' recommended treatment options except in cases when these are preferred by the owner/referring veterinarian. In agreement with our study, previously published data have highlighted that therapeutic shoeing together with controlled exercise had only a 13% success rate for treating chronic HPSD (1). Controlled exercise is often combined with electrophysical therapies such as ESWT, laser, and low-frequency ultrasound therapies (4, 6, 26, 27). Long-term studies investigating ESWT or radial waves have reported fair to poor long-term outcomes (4, 6). In contrast, recent studies using class IV laser and low-frequency ultrasound therapy have reported low re-injury rates (26, 27). However, these latter studies did not mention the number of fore- vs. hind-limb suspensory ligaments nor did they make a distinction between acute and chronic HPSD. Both these factors are crucial for assessing treatment outcomes. Moreover, no control groups were included in either of these studies.

In the current study, the clinical outcomes for the LR-PRP group were superior to those of the control group. To our knowledge, only two large studies have addressed the efficacy of PRP in HPSD (6, 10). However, the percentage of sound horses following treatment for chronic HPSD was not clearly mentioned. These former studies used either single- or multi-intra-lesion injections, while in the current study, only a single injection of LR-PRP was used. Disparities in therapy regimes and in concentrations of platelets and leukocytes among different LR-PRP products might affect the results (9, 10, 23). The leukocyte concentrations in autologous platelet products are categorised as either leukocyte poor (LP) or leukocyte rich (LR) (28). There is much debate in the literature regarding the efficacy of LP-PRP vs. LR-PRP to treat tendinopathy and desmopathy (12, 13, 28). The selection of LR-PRP in the present study was based both on the simplicity of the method involved to recover the platelets even in non-clinical facilities as well as on previous studies in favour of LR-PRP (29). It is presumed the chemotactic stimuli from neutrophils attract macrophages, whose presence contributes to the enhancement of repair mechanisms (30). Additionally, the interaction between neutrophils and platelets leads to the release of anti-inflammatory molecules (31). It has been previously shown that LR-PRP provides a higher concentration of transforming and platelet-derived growth factors (TGF-1, PDGF) (23, 31) as well as vascular endothelial growth factor (VEGF) (32), which are essential for neovascularisation and extracellular matrix synthesis (8). Increase of the concentration of growth factors in PRP can be achieved by using ESWT directly after the injection or by freeze-thaw cycle (33). However, patients with PRP and ESWT were excluded from this study, since additional treatments could have obscured the evaluation of PRP treatment response, and activation with a freeze-thaw cycle does not appear to result in improved tendon healing compared to the use of fresh PRP (34).

BMAC treatment is a one-step, non-surgical, cost-effective and minimally invasive procedure which can be safely executed by an experienced clinician at the point of care (24). Although the mechanism of action is not fully understood, numerous preclinical and clinical studies in laboratory animals and humans have reported that BMAC treatment yields promising outcomes in tendinopathy and desmopathy (18, 35, 36). In horses, experimental and clinical models suggest that the anabolic effects of acellular BMA and bone marrow-derived mononuclear cells (BMMNCs) occur by increasing the collagen I/III ratio and the expression of cartilage oligomeric matrix protein (17, 37, 38). BMMNCs are heterogeneous cell populations including stem cells (SCs), endothelial progenitor cells, monocytes and lymphocytes (39, 40). A synergistic effect among the heterogeneous cell populations promotes neovascularisation even in previously avascular tissue (39, 41, 42).

In the current study, the concentration of platelets in the LR-PRP treatment (median 495 × 10^6^/mL) (23) was higher than that in the BMAC treatment (median 160 × 10^6^/mL). Furthermore, quantitative analysis of growth factors in a previous study revealed that TGF-1, PDGF, and VEGF are present in greater concentrations in LR-PRP than in BMAC produced by commercial kit (43). Nevertheless, the LR-PRP treatment was significantly inferior to the BMAC treatment, which was in agreement with a previous *in vitro* study (36). Hence, the presence of high platelet concentration in PSL may not be the sole efficacy mechanism in the BMAC group. BMAC should not be considered as pure stem cell therapy, since SCs have a poor concentration in BMAC, but rather as a mixture of regenerative heterogeneous cells, bioactive molecules, growth factors and platelets. Even more, nucleated cells from fresh BMAC have different surface markers in comparison with those from cultured BMA-mesenchymal stem cells (44) and *in vitro* studies, fresh BMAC cells have yielded better results than cultured BMA-mesenchymal SCs (45). Therefore, the authors of this study theorise that the synergy of all above-mentioned parts constituted BMAC promotes a stable microenvironment and provides strong stimuli for biosynthesis, downregulation of inflammatory process, angiogenesis, and fibroblast proliferation. The additional presence of platelets in BMAC may augment angiogenesis and proliferation of regenerative cells (46). Similar assumptions can explain the insignificant differences between lame and sound horses in terms of BMAC platelet, monocyte, or WBC counts, although a wide range of the above-mentioned values was observed among the BMAC samples. Another possible explanation would be that other elements of BMAC, like cytokines, which were not measured in this study, are crucial for the healing of the PSL.

**Figure 9:**
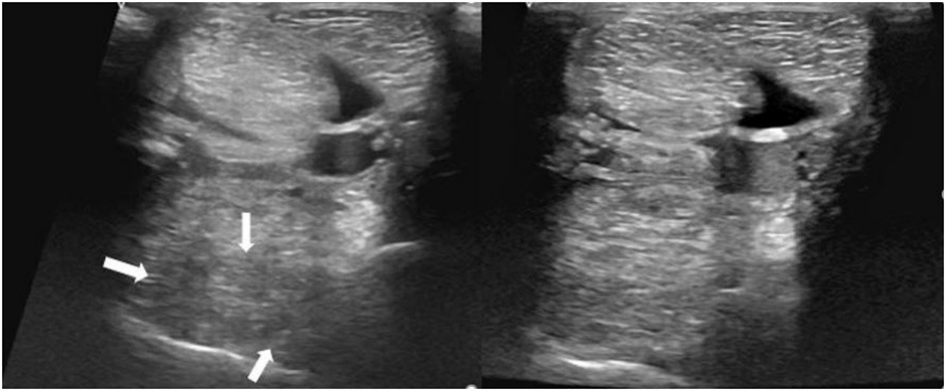
Transverse images of a proximal suspensory ligament about 5 cm distal the head of the MTII. Left is before the treatment and right ~6 months after the treatment with BMAC. The lesion is distinguished by three hypoechogenic areas (white arrows), loss of the dorsal margins and enlargement of the ligament. Despite the obvious increase of the echogenicity and reduction of the size of the proximal suspensory ligament, this horse never became sound.

The use of the ultrasound modality for establishing the diagnosis of HPSD and assessing the healing process during the rehabilitation phase is very common in clinical practise (1–5, 9, 14, 22, 47). The absence of ultrasonographic improvement was correlated with the recurrence of HPSD in this study, independent of the previous treatment. Cases of persistent or recurrent lameness despite ultrasound improvements (Figure 9) in the BMAC and LR-PRP groups could have resulted from compartment-like syndrome, formation of adhesions, osseous pathology undetectable with radiology, or neuropathy of the deep branch of the lateral planter nerve (48–51). It could be argued that clinical outcomes depend upon lesion severity (1, 6), this possibly suggesting that ultrasound scores at the initial examination may contribute to the long-term prognosis following orthobiologic treatment for HPSD (Table 4). In the present study, the authors used a grading scale for the ultrasound lesions, as is common in most HPSD studies (4, 9, 14, 24). However, ultrasound examination alone can be an unreliable method to determine the exact severity of a lesion since the gold standard diagnostic modality for hindlimb PSL is the high field MRI examination (51–55). Additionally, using only radiology and ultrasound for hindlimb PSL examination, osseous pathology and neuropathy of the deep plantar nerve can be failed to be detected in concurrent HPSD (51, 53).

**Table 4:** Ultrasound scores among the groups at the presentation days (T0).

**Group**	**Lesion 1**	**Lesion 2**	**Lesion 3**	**Lesion 4**
PRP, considered sound/total (%)	10/17 (47.6)	8/18 (44.4)	2/7 (28.6)	0/4 (0)
BMAC, considered sound/total (%)	7/8 (87.5)	7/9 (77.7)	3/4 (75)	1/4 (25)
Control, considered sound/total (%)	1/9 (11.1)	0/7 (0)	0/3 (0)	0/3 (0)

*Within the parenthesis is the percentage of horses considered sound at the evaluation 18 months after the treatment*.

An assessment of clinical outcomes <6 months post-treatment was not included in this study, since most recurrences of HPSD occur after a return to normal training (14). Thus, long-term (>1 year) results are more reliable to evaluate chronic HPSD treatment efficacy (1, 4, 9, 14, 48). The selection criteria in this study were established in order to reduce clinical heterogeneity and maximise statistical consistency by evaluating long-term treatment effects in a single lesion type. Further effort was made to reduce operator variability. Therefore, the clinical evaluation, ultrasound performance and course of treatments were carried out by a single clinician based on a standardised clinical protocol at the clinic. The majority of HPSD cases were dressage horses, which is not surprising given that the repetitive collection exercises in this discipline predispose the horses to chronic hindlimb PSL injury (2). Although the Andalusian horses with HPSD tend to have limited response to therapy due to their common up-right hock angle conformation (47, 48), there was no significant difference in distribution of this breed among the groups. Another important variable in this study population was the level of performance, which ranged from a national low-level to international high level. However, there was an equal distribution of high-level performance horses in the LR-PRP and BMAC groups, whereas rather middle- and low level of performance horses tended to be enrolled in the control group. Many moderate and high level of performance horses could return to their previous performance with modified training, for example, 1 day with training—1 day without. The authors in this study did not evaluate the association between age and return to previous performance after each treatment within the groups of LR-PRP and BMAC, since the samples were too small.

One limitation of the current study is the absence of MRI examination, since sonography has lower sensitivity and specificity for detecting HPSD compared to high field MRI (51–55). However, the high cost of the MRI examination and the risk of general anaesthesia were the most common limiting factors of using MRI in horses with HPSD in this study. Despite the fact that ultrasonography is an operator-dependent modality, the comparison with the gold standard histopathologic examination has revealed high reliability of detection HPSD (49). Nevertheless, the authors in this study excluded patients with positive PSL diagnostic analgesia and normal radiographic and ultrasonographic findings, since specificity (true negative) in detection HPSD with these diagnostic modalities compared to high field MRI is low (51, 53). Another limitation is the selection of patients without considering limb conformation since a straight tarsal angle and/or hyperextension of the fetlock joint is a predisposed factor for HPSD (47, 56). However, warmblood horses with this conformation are withdrawn from the riding career by the authors after HPSD, since according to the authors' experience an in agreement with previous literature (47, 48) the risk of HPSD recurrence and/or additional injuries in the suspensory ligament branches is high. Therefore, these patients had to be excluded from the study population, as they did not have follow-up. Moreover, this factor should be considered in the light of the retrospective nature of this study. Additional limitation was the non-blind clinical evaluation at T1 by the first author, which can create observation bias in this study. In order to overcome this bias all referring vets were called to assess the lameness at T2 and T3. Another variable for the assessment of the treatment's outcome was the percentage of horses returning to previous performance, since this variable enhances the reliability of the long-term results after each treatment. The percentage of horses considered sound at evaluation by the first author at T1 and by the referring vets at T2 and T3 as well as the percentage of horses, which returned to previous performance at T2 and T3, both have showed similar significance among the groups. Finally, cytology profiles were not analysed in LR-PRP since the authors relied on the results of previous studies (20). Although, measurements of growth factors and cytokines were not performed in either the LR-PRP or BMAC samples since all cases were treated under private practise circumstances, knowledge of these concentrations would not have influence on the results, since the groups were formed according to the biological treatment and not according to the cytokines or growth factors concentrations.

In conclusion, a single intralesional treatment with PRP or BMAC in chronic HPSD showed better improvement in ultrasonographic scores, lameness grade and return to previous activity level than the controlled exercise alone treatment. BMAC may be considered as an advanced non-surgical treatment option in >3 months HPSD with satisfactory long-term results in sports horses.

## Data Availability Statement

The raw data supporting the conclusions of this article will be made available by the authors, without undue reservation.

## Ethics Statement

Ethical review and approval was not required for the animal study because it was a retrospective study. Written informed consent for participation was not obtained from the owners because it was a retrospective study.

## Author Contributions

GM was responsible for the study execution and data collection. GM and MM were involved in study design, data analysis, and interpretation and preparation of manuscript. All authors contributed to the article and approved the submitted version.

## Conflict of Interest

The authors declare that the research was conducted in the absence of any commercial or financial relationships that could be construed as a potential conflict of interest.
